# Supplementary material for: Neural networks for self-adjusting mutation rate estimation when the recombination rate is unknown
Source: PLoS Comput Biol. 2022 Aug 3;18(8):e1010407. doi: 10.1371/journal.pcbi.1010407 (PMC9377634; doi:10.1371/journal.pcbi.1010407)
Supplement: Attachment — Submitted filename: AnswerToReviewers.pdf [file pcbi.1010407.s002.pdf]

Title:
Neural Networks for self-adjusting Mutation Rate Estimation when the Recombination Rate is unknown

Authors:
Klara Elisabeth Burger, Peter Pfaffelhuber, Franz Baumdicker

Dear reviewers,
thanks for your careful reading of the manuscript and the helpful comments and suggestions. We are confident we could address all of your comments and hope to have clarified all open issues. In particular, we have added a more realistic scenario, where we applied out method using the recombination map of a human chromosome. This shows that our method is working in a realistic parameter regime for real data applications. Furthermore, we added a comparison with two sophisticated methods, namely the CNN proposed by Flagel et al. and an ABC analysis. In addition we looked at the feature importance of the neural network and could thereby highlight that the neural network learned to distinguish different recombination rates. We believe your comments and suggestions enabled us to significantly improve this manuscript. Below is a detailed list of answers to the points you raised.

Reviewer 1
In this study, authors propose to estimate the population mutation rate ($\theta = 4N_e\mu$) from the site frequency spectrum using neural networks. They compare the performance of their estimation against known estimators at several experimental conditions. The idea is of interest and it aligns with several ongoing efforts to use neural networks to estimate population genetic parameters.
Answer: Thanks for your kind words.

I have some comments.

Q: How would other deep learning algorithms that have been proposed recently compare against the one proposed in this study to infer the mutation rate? Also, since training is done by simulations, how would ABC perform in this case? It would be of interest to assess how much a simpler approach proposed here (MLP with 0 or 1 hidden layers from the SFS) compare against more sophisticated methods (e.g. CNN or RNN or GANs from either summary statistics or raw genomic data). In particular, how does it compare against Adrion et al. 2020 MBE? In general, it seems that more complex architectures have been proposed in popgen to infer more challenging parameters (e.g. full demographic histories rather than one estimate of theta). Therefore, it should be more evident the contribution to the field that this paper brings.

A: *We think this is a very interesting question. Originally we were planning to look into this in more detail in a separate project. But you are right, the current manuscript also benefits from a comparison with more sophisticated methods. Thus we tried multiple alternative more complex methods and checked whether a comparison to our method would be reasonable. We identified two methods that can act on the same parameter regime. Namely the CNN proposed by Flagel et. al, as well as an ABC approach. A comparison with both methods has been integrated into this manuscript.*
We considered other methods, which did not turn out to be applicable to our scenario. The suggested method from Adrion et al. 2020 "Predicting the Landscape of Recombination Using Deep Learning" was trained to learn the recombination rate, while our method estimates the mutation rate. We really hoped to compare our method to the combination of convolutional neural networks with ABC suggested by Sanchez et. al. 2020: "Deep learning for population size history inference: Design, comparison and combination with approximate Bayesian computation". Unfortunately the architecture of the method required larger genotype matrix data than our parameter regime would create. For both methods a consideration would thus

only have been possible if we redesigned the architecture of those networks, which is beyond the scope of this manuscript. However, the comparison to ABC and a - to our parameter regime retrained - CNN suggested by Flagel is now part of the manuscript and significantly improved it. Thanks for this comment.

Q: To clarify the scope of this study, it would be useful to deploy the train network to real data to infer the mutation rate on species/populations of interest to gain some biological insights.

A: *We have added an application to datasets where the HapMap recombination map of the human chromosome 2 was used to generate more realistic site frequency spectra along a chromosome. This allowed us to highlight that what we consider as low, intermediate, and high recombination rates in this manuscript can indeed occur in real data and that our method provides a robust estimator in this scenario with non-constant recombination rates. An additional application to real data would be possible in principle, but in our opinion would distract the reader too much from the main message. We believe the more realistic scenario now included gives a good impression of the applicability of our method to real data.*

Q: I could not find details on the division of simulations between training, validation and testing data sets. Is the performance shown coming from the testing set? Also, can you show that you don't have overfitting? How did you do hyperparameters tuning?

A: *We added a section with details to appendix B. The performance was evaluated on the test set. In addition, a plot showing the performance of the neural networks outside of the training range, can be found in the appendix as well.*

Q: Regarding simulations, what is the unit of measure for the recombination rate? Is it scaled by N_e? What is the length of the region simulated? Would it be more realistic to simulate variable recombination rates that mimic known recombination maps (e.g. in humans).

A: *Yes the recombination rate is scaled by N_e. We added an explanation in (line 140 ff). As long as we are only interested in the SFS the scaled recombination rate suffices and we did not specify the length of the simulated region. This changed in the now novel application using the human recombination map of chr2. See also our answer above.*

Q: The introduction and literature review are too succinct in my opinion. For instance, I was expecting more information of the biological context of the study: What is the mutation rate in various domains in life? How is regulated? How does it vary along the genome or between populations? What is the importance of inferring this value? Additionally, there are more papers using deep learning in population genetics and authors should also briefly survey the different architectures and input data used by these previously published studies.

A: *We agree and extended the introduction and literature review significantly. We included a more detailed background on mutation rates, but also the effective population size, as estimating N_e is often equivalent to estimating the mutation rate. In addition, we shortly mention different architectures and input data, including the genotype matrix and ancestral recombination graphs.*

Q: Figure 2 is a cartoon of a generic MLP and it seems to me that it doesn't provide any specific information. Likewise, Figure 1 is an illustration for calculating the SFS from a coalescent tree and it appears rather simplistic. I also wonder whether Figure 3 should be better presented as a formal algorithm, given the audience of this journal.

A: *We agree and have moved the Figures 1 and 2 to the supplemental Figures. Figure 3 is now presented as formal algorithm (algorithm 1 in the manuscript).*

Q: From Figure 4, it is clear that neural nets provide better estimates at moderate recombination rate, but what is the scale of this improvement? In other words, and for instance, how much biased downstream analyses (e.g. N_e) would be when using classic estimators instead of neural nets? This should give a better idea of the impact of the deep learning approach.

A: *The estimate of N_e is proportional to the mutation rate estimate and estimates of N_e would thus be affected exactly in the same way as the mutation rate. We have extended the introduction section with a detailed review of the importance of N_e and its relationship to the mutation rate. In addition, we have added a supplemental figure using a recombination map with regions of low and high recombination rates that shows the difference between mainly Watterson's estimator and the more robust nerual network based estimation on local estimates of the effective population size and the mutation rate.*

Q: It should be made clearer in the methods what is novel and what is already know. From my understanding, everything from line 58 to 94 are derivations already known.

A: *This is correct. To clarify, the following passage has been added to the manuscript: "The model-based estimators presented below were proposed and analyzed by Watterson [14], Fu [15] and Futschik et al. [16]. In order to give a self-contained presentation, we now recall some basics on the coalescent and give details of the above estimators:"*

Additional points:

- It would be of interest to comment on the inference of other estimators of the population mutation rate based on gene diversity (rather than S) and their various extensions (as in https://academic.oup.com/mbe/article/26/3/501/976423), and how neural nets can tackle this problem.
We have added a short corresponding statement to the discussion in line 333 ff.
- Can you infer the optimal estimator for the trained neural nets? In other words, from the learned weights can you attempt any interpretation of what are the most important input units (the SFS) and their combination for the parameter to predict?
Thanks for this helpful comment, this is a very good question. Inferring the coefficients itself is not directly possible, even for the linear neural network, as the nn are trained for variable theta. But we have followed up on this and have attached a section to appendix A. There the feature importances of both linear neural network and adaptive neural network are considered using the tool SHAP. We believe this significantly improved our manuscript.
- In the abstract, "For intermediate recombination rates, the calculation of optimal estimators is more involved", do you mean "is more convoluted" or "challenging"?
Changed to more challenging.
- The very first sentence is too long and convoluted for being the beginning of a paper.
Thank you for this helpful comment, we have rephrased and split the sentence into multiple sentences.
- Line 49: I'd say that there isn't a limited theoretical insight in population genetics.
We agree, the sentence has been rephrased.
- Line 113: n is number of chromosomal copies or diploids?
It is the number of haploids, we have clarified it in line 139 as well.
- Is there any scope to jointly infer mutation and recombination rates?
We hypothesize that it might be possible to estimate recombination rates with a similar architecture. This might be an interesting follow up project.
- Is it possible to do an exhaustive architecture search to find the optimal set up for this study?
We have added a section on the choice of number of nodes. (lines 165 ff) An exhaustive architecture search was outside the scope of this study.

Reviewer 2

This paper by Burger et al. develops some fairly simple artificial neural networks as alternatives to model-based estimators of theta. This is a relevant topic because existing estimators do well in the case of free recombination or no recombination, but struggle in intermediate cases. The authors show that their neural network approach yields a more general-purpose estimator. I found this paper interesting and well-written, and have only a number of relatively minor comments that I would like to see addressed prior to publication:

Answer: Thanks for your efforts and your positive review.

Comments:

Q: Line 138: "It is advisable to use at least 2n nodes." It would be more helpful if the authors could share any analyses that support this claim.

A: *To clearify, we added a section in line 165 ff.*

Q: Between Lines 150-151: Can the authors explain how t_k are chosen? (Referring the reader to Figure S4 might also help here somewhat, but this Figure is not currently referenced in the text.)

A: *Thank you for the helpful comment. We agree and have rephrased the corresponding section and added a reference to Figure S4.*

Q: Also, the condition that the authors are trying to satisfy when choosing the t_k includes the term $a_j(t_{k-1})$. When $k = 1$, this gives $a_j(t_0)$, but I don't think this is defined.

A: *Thanks for spotting this! We did actually not rely on the value of $a_j(0)$ in our class definition and changed the corrsponding description.*

Q: Line 155: The authors state that training finishes only once the network performs "comparably or better than the model-based estimators and the linear NN on each of the six test subsets." I have two comments on this: 1) By comparable or better, the authors mean "exactly equal to or better", correct? 2) Is this always guaranteed to happen? I would imagine that, especially if one were to test a neural network with low capacity, the network may not always be able to achieve this. Did this ever happen when the authors were testing out different architectures? I think some readers would find this information very useful and interesting.

A: *Thanks for this excellent question.*

1) *You are right, we have not been clear at this point. With "comparable or better" we mean "better or only slightly worse". We have added the precise definition of this condition to the manuscript (Equation 3). Thanks for this comment!*
2) *This is not guaranteed as there is no formal proof of convergence. You are right, the convergence of the neural network strongly depends on its capacity. If one chooses to few hidden nodes(i.e. less than $n = 40$), the neural network is likely to not terminate. This is why we recommend to use at least $2n$ hidden. With this rule of thumb we have not observed any issues. A section explaining this linkage has been added to the manuscript.*

Q: Line 179: The authors kind of downplay the performance of the linear neural net here. Looking at the results, it seems that this simple network has done quite well! I would consider rephrasing the text here to more accurately describe these results.

A: *You are correct. Looking at the whole range of possible recombination rates the linear neural network has found a compromise between high and low recombination rates, that would still be a good choice for unknown recombination rates. We have rephrased this part in the manuscript.*

Q: Lines 203-205: Here the authors state that the neural nets "dramatically over or underestimate the mutation rate" outside of the training range of this parameter. But the performance in S2 Fig does not look too bad to me. It seems like the neural nets perform about as well as the best model-based estimator for each case. So, while the error rates increase, it doesn't seem that there is some dramatic collapse once we get outside of the training range, unless I am missing something. Perhaps less pessimistic language would be appropriate here.

A: *Thank you for this comment. It was intended to make this statement more general, as this is often an issue with neural networks. As you correctly observed, it is less of a problem in our case. The corresponding section was adjusted.*

Q: In any case, it is clear that error rate does increase as we move further and further from the training range. What should one do about this if for some population we really have no idea what theta is? How would the authors deal with this in practice?

A: *We have added the following sentence to the discussion section: In an application, if there is no idea about the realistic range of θ, we would recommend first using the Watterson estimator to get an approximate idea about the range of θ and retrain the neural network accordingly.*

Additional points:

- Figure 4 caption: "For each shown data point 10000 simulations with sample size n = 40..." How many data points are shown? This would help the reader determine how many total test simulations were generated for this figure.
  Thank you for this thoughtful comment. In this figure, 49 *data points are shown, resulting in a total test set size of* $4.9 \cdot 10^5$ *simulations. The caption has been adjusted, it should be more clear now.*

- Line 213: What is a standard laptop? Please share the specs if you don't mind.
  Specs are added in line 335.

Reviewer 3
The authors consider the classical problem of estimating the scaled mutation parameter in a population genetic context. They consider feedforward neural networks and show that they are able to achieve a performance comparable to previously proposed estimates both for high and low recombination rate. Indeed, the results seem to indicate that neural networks are able to adapt to an unknown recombination rate, whereas with classical estimators, the correct one has to be picked depending on recombination. Interestingly, the neural networks work well, even without a hidden layer. The obtained results are interesting and contribute to an understanding of potential benefits of machine learning methods compared to mathematical theory based approaches.
Answer: Thank you for your positive review.
Comments:

Q: On the other hand, in practical applications there is usually an idea about the underlying recombination rate. (And if not it can also be estimated.) Therefore the actual gains achieved by machine learning in this context remain unclear. There seem to be some slight advantages of neural networks at intermediate recombination rates, but it would be nice to see a biological example, illustrating that there is actually an organism and a segment length for which the intermediate recombination (and mutation) rates are realistic. It would also be interesting to consider more complex scenarios and see whether neural networks provide larger advantages there (maybe with demography?).

A: *Besides introducing a new estimator for the mutation rate that can be used without knowing the recombination rate we hope the manuscript also highlights the potential of using an adaptive training process based on known classical estimators as a sort of guiding system to*

identify the regions of the training data where an improvement of the neural network is still possible. However, we agree with your point and included a biological example that illustrates that for example for the human chromosome 2 and a window size of 70kb intermediate recombination rates are realistic. However, as the recombination rates varies this is also true for many other window sizes. See also our answer to reviewer 1 for more details. We have included a more complex scenario using a HapMap recombination map instead of constant recombination rates. A study of models including a complex demography is beyond the scope of this study, but we now cite recent publications addressing demography with machine learning tools in the introduction.

In general, the manuscript is well written and structured.

Further comments:

- The authors claim in the abstract and at other places that the Fu's estimate is optimal. This is not correct: Fu shows optimality (BLUE, best linear unbiased estimate) only for a variant of his estimate that requires knowledge of the true unknown mutation rate. But if the mutation parameter is known, there is no need to estimate it anymore. The variant where an estimate of theta is used instead is neither linear nor unbiased. And as far as I know, there is also no proven optimality result for this estimate. The corresponding claims should be rephrased accordingly.
This is correct, optimality is only shown for the estimator requiring the true but unknown θ. Therefore, this estimator is of no practical use, but knowledge of this estimator is still valuable. In particular, as the performance of the iterative estimator that is used when theta is unknown is very close to the performance of the optimal estimator. Nonetheless, we agree with your point that we should be more careful about the phrasing in order to prevent misunderstandings. We have adjusted the abstract and the corresponding sections.

- It is possible to apply estimates on DNA segments short enough so that recombination can be ignored. For constant recombination, these local estimates can be averaged. How does such a strategy work in the case of intermediate recombination rates.
We are not completely sure we understand the proposed strategy correctly. If you suggest to use a smaller window size along the chromosome to decrease the underlying recombination rate we do not think that this would yield a better estimator as smaller values of θ are particularly hard to estimate which motivated us to use the adaptive training procedure in the first place. Somewhat related we have included an artificial block recombination map application in the supplement of the revised manuscript. In this recombination map the recombination rate is locally constant. The application highlights that estimates for neighbor windows in regions with low recombination are correlated.

Own corrections:

- We changed some minor mistakes and typos see diffs.pdf for details.
