# Supplementary material for: Neural networks for self-adjusting mutation rate estimation when the recombination rate is unknown
Source: PLoS Comput Biol. 2022 Aug 3;18(8):e1010407. doi: 10.1371/journal.pcbi.1010407 (PMC9377634; doi:10.1371/journal.pcbi.1010407)
Supplement: Attachment — Submitted filename: CommentsForReviewer2.pdf [file pcbi.1010407.s003.pdf]

Title:
Neural Networks for self-adjusting Mutation Rate Estimation when the Recombination Rate is unknown

Authors:
Klara Elisabeth Burger, Peter Pfaffelhuber, Franz Baumdicker

Dear reviewers,
thank you for your positve review of our revised version and further helpful comments and suggestions. We addressed all of your remaining comments.
We believe that your comments and suggestions have allowed us to improve this manuscript once again. Below is a detailed list of responses to the final points you raised.

Reviewer #1
Authors now compare their method with competing strategies. Whilst they do not deploy the network to real data, they use realistic recombination maps for their simulations. The introduction is now more comprehensive. Details or explanations that were missing are now present. Minor things have been fixed as suggested. The manuscript has significantly improved.
Answer: Thanks for your kind words.

Comments:

Q: As a minor comment, I'd suggest the Authors to consider colourblind-friendly colour-palette (e.g. for Fig 6).

A: *We agree, and all plots are now displayed in a colourblind-friendly colour-palette.*

Q: Also the title "comparison to more sophisticated ML Methods" can be changed to simply "comparison with alternative/competing/existing ML methods". Is ABC an ML method? In case, the "ml" in the title can be dropped.

A: *Thank you for this comment. The title has been changed to "Comparison with Alternative ML Methods".*

Q: In Fig 5 different lines are hard to see.

A: *We agree. The lines would be more visible if we were to show only a section of the figure. However, this would limit the comparability with the recombination map in Fig 4. Since we believe that the figure nevertheless emphasizes the main message, we have not adjusted it, but have included an extract from Fig. 4 and Fig. 5 in the supplement (Fig. S9 and Fig. S10), in which lines are more visible.*

Reviewer #2
The authors have done a thorough job addressing my (mostly minor) comments, and the paper is suitable for publication as is.
Answer: Thank you for your positive review.

Reviewer #3
This new version of the manuscript is quite pleasant to read. The revision has addressed my concerns.
Answer: Thank you for your positive review.

I have only two minor points.

Q: l. 113-114: What do you mean by not uniform? The actual estimator with estimated theta is neither linear nor unbiased, and there is no proof that it is unbiased.

A: *That is correct, thank you for pointing this out. The corresponding section has been rewritten and should be clear now.*

Q: l. 121-123: There are actually multiple variants of the estimate proposed by Futschik & Gach. One of them is a modification of Watterson's estimate and does not depend on theta. There are also iterative versions, both of Fu's estimate and the Watterson estimate. This should be mentioned and it should be explained more clearly which of the variants has been used. It would be nice to show also the performance of the non-iterative modification of Watterson's estimate, maybe as supplementary material.

A: *This is a good point, we have clarified which variants of the Futschik & Gach, Fu and Watterson estimators are used. A performance plot of the non-iterative modification of Watterson's estimator for* $\rho = 0$ *has been included in the appendix (Fig S8).*

Own corrections:

- We changed some minor mistakes and typos see diffs.pdf for details.
